# COX5A over-expression protects cortical neurons from hypoxic ischemic injury in neonatal rats associated with TPI up-regulation

**DOI:** 10.1186/s12868-020-00565-5

**Published:** 2020-04-29

**Authors:** Ya Jiang, Xue Bai, Ting-Ting Li, Mohammed AL-Hawwas, Yuan Jin, Yu Zou, Yue Hu, Lin-Yi Liu, Ying Zhang, Qing Liu, Hao Yang, Jun Ma, Ting-Hua Wang, Jia Liu, Liu-Lin Xiong

**Affiliations:** 1 Laboratory Zoology Department, Institute of Neuroscience, Kunming Medical University, Kunming, China; 2 School of Pharmacy and Medical Sciences, Division of Health Sciences, University of South Australia, Adelaide, 5000 South Australia; 3 National Traditional Chinese Medicine Clinical Research Base and Western Medicine Translational Medicine Research Center, Department of Cardiac and Cerebral Diseases, Affiliated Traditional Chinese Medicine Hospital, Southwest Medical University, Luzhou, 646000 China; 4 Department of Neurosurgery, The First People’s Hospital of Zhaotong, Zhaotong, 657000 China

**Keywords:** Neonatal hypoxic-ischemic encephalopathy, COX5A overexpression, Neuronal survival, TPI

## Abstract

**Background:**

Neonatal hypoxic-ischemic encephalopathy (HIE) represents as a major cause of neonatal morbidity and mortality. However, the underlying molecular mechanisms in brain damage are still not fully elucidated. This study was conducted to determine the specific potential molecular mechanism in the hypoxic-ischemic induced cerebral injury.

**Methods:**

Here, hypoxic-ischemic (HI) animal models were established and primary cortical neurons were subjected to oxygen–glucose deprivation (OGD) to mimic HIE model in vivo and in vitro. The HI-induced neurological injury was evaluated by Zea-longa scores, Triphenyte-trazoliumchloride (TTC) staining the Terminal Deoxynucleotidyl Transferased Utp Nick End Labeling (TUNEL) and immunofluorescent staining. Then the expression of Cytochrome c oxidase subunit 5a (COX5A) was determined by immunohistochemistry, western blotting (WB) and quantitative real time Polymerase Chain Reaction (qRT-PCR) techniques. Moreover, HSV-mediated COX5A over-expression virus was transducted into OGD neurons to explore the role of COX5A in vitro, and the underlying mechanism was predicted by GeneMANIA, then verified by WB and qRT-PCR.

**Results:**

HI induced a severe neurological dysfunction, brain infarction, and cell apoptosis as well as obvious neuron loss in neonatal rats, in corresponding to the decrease on the expression of COX5A in both sides of the brain. What’s more, COX5A over-expression significantly promoted the neuronal survival, reduced the apoptosis rate, and markedly increased the neurites length after OGD. Moreover, Triosephosephate isomerase (TPI) was predicted as physical interactions with COX5A, and COX5A over-expression largely increased the expressional level of TPI.

**Conclusions:**

The present findings suggest that COX5A plays an important role in promoting neurological recovery after HI, and this process is related to TPI up-regulation.

## Background

Neonatal hypoxic-ischemic encephalopathy (HIE) could impact neuronal development and result in abnormal function in whole life, which may underlie the cause of seizures and other severe neurological deficits in children [1]. As a destructive disease that causes the death of infants [2–4], HIE occurs in 1.5/1000 live births in China [5], and 0.2–0.3% of live births in the United States are recorded with HIE cases. Severely, approximately 25% of HIE infants die in their first year, while another 25% survive but with permanent neurological disabilities [6]. The most significant risk factor for HIE is perinatal asphyxia that may occur either in utero or post-natally. Utero asphyxia is caused by inadequate blood flow or oxygen supply resulting in breathing difficulty and even in breathing arrest altogether. Postnatal asphyxia causes neonatal pulmonary failure such as severe hyaline membrane disease, meconium aspiration syndrome, pneumonia as well as congenital heart disease [7–10]. However, brain damage and neuronal cell death through oxidative stress and excitotoxicity [11] are amongst the outcomes of HIE causing high rate of mortality and morbidity in infants.

Increasing treatment studies targeting neonatal HIE have been developed over the years, and currently, hypothermia has been considered as a standard therapy. Neonates receiving hypothermia go through less death and incidence in IQ score below 70, less severe disability as well as attention-executive dysfunction [12]. However, its limited effectiveness remains incompletely certain [13]. Besides the hypothermia, the current therapies including erythropoietin (EPO) [14], hyperbaric oxygen (HBO) [14], Xenon [15, 16] and melatonin [17] treatments exert protective effects on the brain and peripheral organs following HIE, but less than satisfactory. The main reason for this phenomenon is that the molecular mechanisms underlying cell damage in HIE still remain vague, and there lacks an effective intervention against development of HIE. Thus novel effective interventions based on the molecular mechanisms are urgently needed.

It has been reported that Cytochrome c oxidase subunit 5a (COX5A) combines with COX5b to form the COX5, a subtype of cytochrome c oxidase (COX) related to mitochondria activity [18]. In addition, COX is an endogenous metabolic marker and is closely related to energy metabolism [19]. For example, employing a 4-vessel occlusion model of cerebral ischemia in rats, a previous study revealed the activity of COX was decreased at 1 h after ischemia, which may be associated with the neuronal ischemic injury [20]. Moreover, the investigation was expanded in the ischemic-reperfusion model to further explore the effects of mitochondrial protein synthesis and COX activity, and the results showed that the degree of COX activity was also decreased to 90.3%, 80.3%, 81.9% and 83% of the control values at 15 min after cerebral ischemia and 1, 3, 24 h after reperfusion, respectively [21]. Likewise, another study reported that COX activity was significantly decreased at 3 h after traumatic brain injury, and the COX5A subunit was oxdatively modified in the hippocampus [22]. These studies thus indicated that COX5A may play important roles in the process of central nervous system diseases. However, the role of COX5A and its associated mechanism with neonatal HIE remain to be explored.

Here, in order to find out new targets based on COX5A and provide the basic evidence for the treatment of HIE in future, we utilized the animal model of neonatal HI injury together with primary cortical neurons subjected to oxygen–glucose deprivation (OGD) as in vitro model to analyze the role of COX5A and determine the related molecular mechanisms in HIE insults.

## Materials and methods

### Animals and grouping

Forty-seven-day-old postnatal (P7) Sprague–Dawley (SD) male rats (12–15 g) used for in vivo experiments and 21-day-old rats for primary cortical neurons cultures were purchased from the Department of Zoology of Kunming Medical University. Experimental procedures were reviewed following the standard biosecurity and institutional safety procedures and approved by the Ethic Committees at Kunming Medical University in Yunnan province, China (reference number: kmmu2018031). All animals were raised with their mother in plastic cages with soft wood and free access to food and water in a temperature (21–25 °C) and humidity (50–60%)-controlled room. Experimental operations and data analysis can’t be done by the same researchers, and data analysis must be dealt by at least two researchers blinded to the experimental design.

### Rat model of neonatal HI injury

In this experiment, we employed the classic Rice-Vannucci method to establish the HI model [23, 24]. Briefly, P7 SD rats were anesthetized with isoflurane (4% for induction, 2% for sustained inhalation anesthesia) after they were weighed and numbered. The hypoxic chamber was set at 37 °C with humidity 50–80% before the operation. Briefly, the midline of the ventral cervical skin was cut followed by a blunt dissection of parenchyma, to expose the right common carotid. Subsequently, the right common carotid was ligated by an electric coagulator. Then, the wound was stitched and animals were returned to their mother for 1 h before being kept in a hypoxic chamber under 8% O_2_ and 92% N_2_ (flow rate 3 L/min) for 2 h. Rats in the sham group underwent the same procedures, apart from ligation of the right carotid artery.

### Zea-longa score

Zea-longa scores were used to evaluate the neurological function in neonatal rats subjected to HI injury, verifying the successful modeling. All rats underwent the zea-longa score test to evaluate the neurological dysfunction at 0 h, 2 h, 4 h, 8 h, and 16 h after HI. The specific scoring criteria were as follows: 0 points—no signs of nerve injury; 1 point—the contralateral forepaw lost ability to become fully stretched; 2 points—animals turns to one side while walking; 3 points—walking is unstable, falling to one side; 4 points—loss of consciousness [25].

### Tissue acquisition

For morphological analysis, rats in the sham (n = 5 for TTC staining, n = 3 for immunohistochemical and immunofluorescent staining) and HI (n = 5 for TTC staining, n = 3 for immunohistochemical and immunofluorescent staining) groups were euthanatized at 16 h after HI under deep anesthesia with 4% isoflurane (sustained inhalation anesthesia) for 2 min. Then, after the perfusion of 0.9% normal saline followed by 4% paraformaldehyde, the brain was harvested and put into 4% paraformaldehyde for more than 72 h. With paraffin embedded, the brain Sects. (5 μm) were prepared for immunohistochemical staining and immunofluorescent staining, and the core infarction regions were the key observations.

For molecular biology analysis, rats in the sham (n = 6 for qRT-PCR and n = 6 for WB) and HI (n = 6 for qRT-PCR and n = 6 for WB) groups were sacrificed at 16 h after the surgery under deep anesthesia by continuously inhaling 4% isoflurane for 2 min followed by perfusion with 0.9% normal saline. Thereafter, the white infarcted area and non-infracted area of the contralat-eral hemisphere were removed and stored at -80 °C for further WB and qRT-PCR.

### Triphenyte-trazoliumchloride (TTC) staining

To evaluate the brain damages after HI, TTC staining was performed to observe the infarction of brain tissues in neonatal HI rats. The whole brain from the sham and HI rats was quickly removed at 16 h after rats being deeply anaesthetized with 4% isoflurane, brain tissues was taken out (operating on ice) and frozen in a refrigerator at − 20 °C for 10 min before being cut into five pieces (2 mm each). Afterwards, the sections were placed in 1% TTC solution at 37 °C and incubated for 30 min. The sections were then captured using the digital camera. The non-ischemic necrotic area was pale red, and the ischemic necrotic tissue was white. The ratio of infarct was analyzed using Image J software [26].

### Immunohistochemistry in vivo

The immunohistochemistry was employed to detect COX5A expression. The collected brain was paraffin-embedded and cut into 5 µm sections, then fixed on the glass slides. Before immunohistochemical staining, the sections were deparaffinized and a circle was drawn around the tissue with a PAP pen. Thereafter, sections were washed for three times with 0.01 M PBS buffer (PH = 7.45), 5 min each. Next, 3% hydrogen peroxide was added onto the sections followed by incubating at 37 °C for 15 min and washed as described above. In order to block the non-specific binding in the tissue, a drop of 5% goat serum (Solarbio, S9070) was added on the section and incubated at 37 °C for 30 min. Subsequently, the primary antibodies (COX5A, Zhongshanjinqiao, rabbit, 1:50) were added and 2% goat serum was used as the negative control, lefting overnight in a refrigerator at 4 °C. The next day, sections were washed with 0.01 M PBS buffer for three times, followed by addition of enhancement solution, incubation at 37 °C for 20 min and washing with 0.01 M PBS three times. Subsequently, the secondary antibody (Zhongshanjinqiao, goat anti-rabbit IgG) was added and the sections were incubated at 37 °C for 1 h, and then washed with PBS. Next, DAB staining was performed for 5 min following the manufacturer’s protocol. To perform nuclei staining, the sections were treated with hematoxylin solution for 5 min, and soaked in 1% HCl-ethanol solution for 10 s followed by 5% ammonia treatment for 1 min. Afterwards, they were dried with serial dilutions of ethanol (75%, 80%, 85%, 95%, and 100%) and treated with xylene solution for 2 min, finally sealed with neutral resins. The immune-positive pictures were captured using a light microscope (Leica DMI 6000 B inverted microscope, Germany). For quantification, three sections of each brain with 50 μm apart were selected, and five fields of each slice were randomly collected at 100×. The positive cells (dark brown labeled) in each field were quantified via Image-Pro Plus 6.0 software (MediaCybernetics, Silver Spring, MD, USA).

### Primary cortical neuron cultures and COX5A-over-expression HSV vector transduction

COX5A overexpression transduction was conducted for the further functional verification of COX5A’s role in the process of HI. The primary cortical neurons were isolated and cultured from freshly dissected brains of rat pups at postnatal day one. In brief, the cortical tissues were dissociated to disperse the cells in a neuro-basal medium (Gibco, California-USA) supplemented with 2% B27 and 0.5 mM of glutamine. The cells were plated on poly-l-lysine and laminin coated vessels at the density as described above and were incubated at 37 °C with 5% CO_2_. The media were then replaced on alternating days as described before [27]. Thereafter, a low toxicity HSV-COX5A virus was purchased from Sky Bio (Beijing-China) to explore the function of COX5A according to the manufacturer’s protocol. Briefly, HSV-COX5A virus was transducted into the neurons after 5 days of incubation at a MOI = 10. GFP, emitting green fluorescence, expressed from a co-infection with a GFP-expressing virus in neurons after transduction. Then the infection rate was calculated (GFP -positive cells/total neurons). Detection of the green fluorescence and qRT-PCR were employed to demonstrate the successful transduction and over-expression of COX5A.

### Oxygen–glucose deprivation (OGD)

After culturing for 7 days, OGD was established to mimic the HI condition in vitro. In detail, following three washes with PBS, the neurons were incubated using glucose-free medium (Gibco, USA) in an anaerobic chamber containing 5% CO_2_ and 95% N_2_ at 37 °C for 90 min as described earlier [27]. Subsequently, cells were returned to original medium, then placed in a normoxic chamber with 95% air and 5% CO_2_ for 16 h before further testing.

### Immunofluorescent staining in vivo and in vitro

In order to examine the neuronal viability after HI injury in vivo and in vitro, immunofluorescence staining was carried out. In vivo, the brain slices (n = 3 brains, and three sections of each brain with 50 μm apart) were subsequently deparaffinized and washed three times with 0.01 M PBS and pre-incubated for 30 min with 0.3% TritonX-100 in 5% normal goat serum. Then they were incubated overnight at 4 °C with primary antibodies against NeuN (mouse, Bioss, 1:100). The next day, the primary antibodies were removed and sections were rinsed with 0.01 M PBS buffer for three times before being incubated with the fluorescence-labeled secondary antibodies of Alexa 488 (anti-mouse, Invitrogen, 1:100) for 1 h at 37 °C. Counterstaining of the nuclei was performed using DAPI staining. As NeuN was located on the cytoplasm and nucleus of neurons, so the positive staining was the nuclei of neurons labeled with green fluorescence and co-stained with DAPI, which showed blue fluorescence. Finally, the slices were observed under a fluorescent microscope (Leica, CM1860, Germany) at 200×. In detail, five fields of each slice were randomly selected for the estimation of relative number of NeuN positive cells (green fluorescence labeled) using Image-Pro Plus 6.0 software.

In vitro, to study the expression of COX5A in OGD condition and the effect of COX5A on Neuronal Class III β-Tubulin (Tuj1^+^) in neurons, the primary neuronal cells from the normal, OGD, OGD + NC, OGD + COX5A groups were cultured on glass cover slips. In brief, the cell sections (n = 6 in each group) were fixed with 4% formalin and blocked with 0.3% TritonX-100 in 5% normal goat serum for 30 min at 37 °C. The slips were then incubated with anti-COX5A (Mouse, Santa Cruz, 1:100) and anti-beta III Tubulin antibody (Mouse, abcam, 1:200) respectively at 37 °C for 16–18 h. Then, the cells were rinsed three times with PBS before being incubated with fluorescence-labeled secondary antibodies of Dylight 594 (anti-mouse, abbkine, 1:200) at 37 °C for 1 h. Then DAPI was used to counterstain the nuclei. Five fields in each well were randomly collected at 400× with a fluorescence microscope (Leica, CM1860, Germany). The COX5A positive cells and average cell number of Tuj^+^ cells, average length of the neurite (about calculating 15 neurons) were quantified by Image-Pro Plus 6.0 software.

### Terminal deoxynucleotidyl transferased Utp nick end labeling (Tunel) in vivo and in vitro

Tunel staining was performed to determine the apoptotic occurrence in the cortical infarction region of neonatal HI rats and cultured primary cortical neurons subjected to OGD. Briefly, the brain slices (n = 3 brains, and three sections of each brain with 50 μm apart) and cell sections (n = 6 in each group) were rinsed three times with 0.01 M PBS buffer for 5 min, and 50 μl of 0.01% sodium citrate and 0.1% TritonX-100 was added onto each slice and incubated for 30 min at 37 °C. Then, the slices were incubated with the tunel mixture reagent (tunel label solution: Tune enzyme solution = 9:1) for 1 h at 37 °C. For the negative control, only the tunel label solution was added dropwise. After that, counterstaining of the nuclei was performed using DAPI staining. Finally, tunel positive cells were labeled by red fluorescence and can be co-stained with DAPI staining, which showed blue fluorescence. For quantification, five fields on each slice around the core infarction regions were randomly chosen for imaging with a fluorescence microscope (Leica, CM1860, Germany) at 200×. Then, about 200 cells in each field were quantified by Image-Pro Plus 6.0 software. Then, apoptosis rate was calculated as (tunel positive cells/DAPI cells) %.

### Quantitative real-time polymerase chain reaction (qRT-PCR)

QRT-PCR was performed to detect the mRNA expression of COX5A and other candidate genes after COX5A over-expression. The primers were designed using Premier 5.0 software, then verified via the BLAST software and synthesised by Takara Bio Inc (Takara, Japan). Total RNAs from the fresh cortex and cultured neurons (n = 6 per group) were extracted using the RNAiso plus kit (TaKaRa, JAPAN) and cDNA Synthesis was performed using the ReviertAid Kit (Thermo Fisher Scientific Inc.). The levels of Gstp1, Sod2, Rho-GDIa, TPI, COX5A and β-actin mRNA expression in the samples were estimated by qRT-PCR (CFX-96, Bio-Rad, USA) according to the manufacturer’s instructions using the primers described in Table 1. Briefly, each reaction was performed in a volume of 20 μl mixture consisting of 10 μl of SYBR Green master mix, 1 μl of cDNA, 7.8 µl of water, and 0.6 µl each of forward and reverse primers. Finally, the data were analyzed using a comparative critical threshold (Ct) method where relative expression was calculated as 2^−∆∆Ct^ method.

**Table 1:** The premier sequences

Factor	Forward	Reverse	Annealing temperature (°C)
β-actin	5′GAAGATCAAGATCATTGCTCC3′	5′TACTCCTGCTTGCTGATCCA3′	52
COX5A	5′GTTGGACCAATCATAGGCGCT3′	5′CAATGTCGATCACATGCACCA3′	56
Rho-GDIa	5′ TACAAGCCTCCAGCCCAGAA 3′	5′ AAGGTCAAGCGGGTCACAAT3′	53
Gstp1	5′ TCAAGGCTCGCTCAAGTCCA 3′	5′ TTGCATCGAAGGTCCTCCAC 3′	52
Sod2	5′ CCCTGACCTGCCTTACGACT 3′	5′ AGCGACCTTGCTCCTTATTG 3′	53
TPI	5′ GGGGCAACTGGAAGATGAA 3′	5′ CCTGGCGAAGTCGATGTAG 3′	52

### Western blotting (WB)

WB was employed with the intent to detect the protein expression of COX5A in the cortex of neonatal HI rats and of TPI in OGD neurons after COX5A overexpression. The right and left cortex from rats of the sham and HI group and primary neuronal cells from the normal, OGD, OGD + NC, OGD + COX5A groups (n = 6 wells in each group) were collected and lysed with RIPA buffer containing a proteinase inhibitor cocktail (Roche) on ice for 30 min, then the lysates were centrifuged at 12,000 rpm for 10 min at 4 °C. Protein concentrations were determined by BCA protein quantification kit. The protein samples were boiled and denatured with a loading buffer and 40 μg of protein was added to each well and run at 120 V for 90 min. After that, the proteins in the gel were transferred to a PVDF membrane at a current of 200 mA. The PVDF membrane was then blocked with 5% skim milk powder for 90 min at room temperature, and further incubated with related primary antibodies (seen in Table 2) at 4 °C overnight. β-actin was used as an internal control. Thereafter, membranes were incubated with the secondary antibodies for 2 h (seen in Table 2). Finally, the membranes were developed and the bands were visualized with the ECL (ECL Western blotting kit) luminescence solution. The quantitative analysis was carried out by Image J software. The data were expressed as a ratio of the protein of interest band to β-actin band optical density values.

**Table 2:** The antibody information of WB

Protein	Manufacturer	Lot number	Dilution ratio
COX5A (primary antibody)	Santa cruz	sc-376907	1:500
β-actin (primary antibody)	Bioss	bsm-33139 M	1:2000
TPI (primary antibody)	Bioss	bs-4042R	1:300
HRP, Goat Anti-Mouse IgG (secondary antibody)	Abbkine	A21020	1:500
HRP, Goat Anti-Mouse IgG (secondary antibody)	Abbkine	A21010	1:500

### Bioinformatics prediction

GeneMANIA provides many large, publicly available biological datasets to find related genes. These include protein–protein, protein-DNA and genetic interactions, pathways, reactions, gene and protein expression data, protein domains and phenotypic screening profiles. Data is regularly updated. The relationship between Gstp1, Sod2, Rho-GDIa, TPI and COX5A was predicted using the GeneMANIA website (http://genemania.org/).

### Statistical analysis

Data were expressed as mean ± standard deviation (SD). The comparison between two groups was performed using a Student’s t test. For comparison of multiple groups in vitro experiments, ANOVA with least-significant difference (LSD) or Dunnett’s T3 post hoc test was applied, if equal variances were found, LSD was performed; otherwise, Dunnett’s T3 was used [28]. All statistical analyses were performed with SPSS18.0 software (IBM Corporation, NY, USA). *p* <  0.05 was considered statistically significant.

## Results

### HI induced cerebral infarction and cell apoptosis

**Fig. 1:**
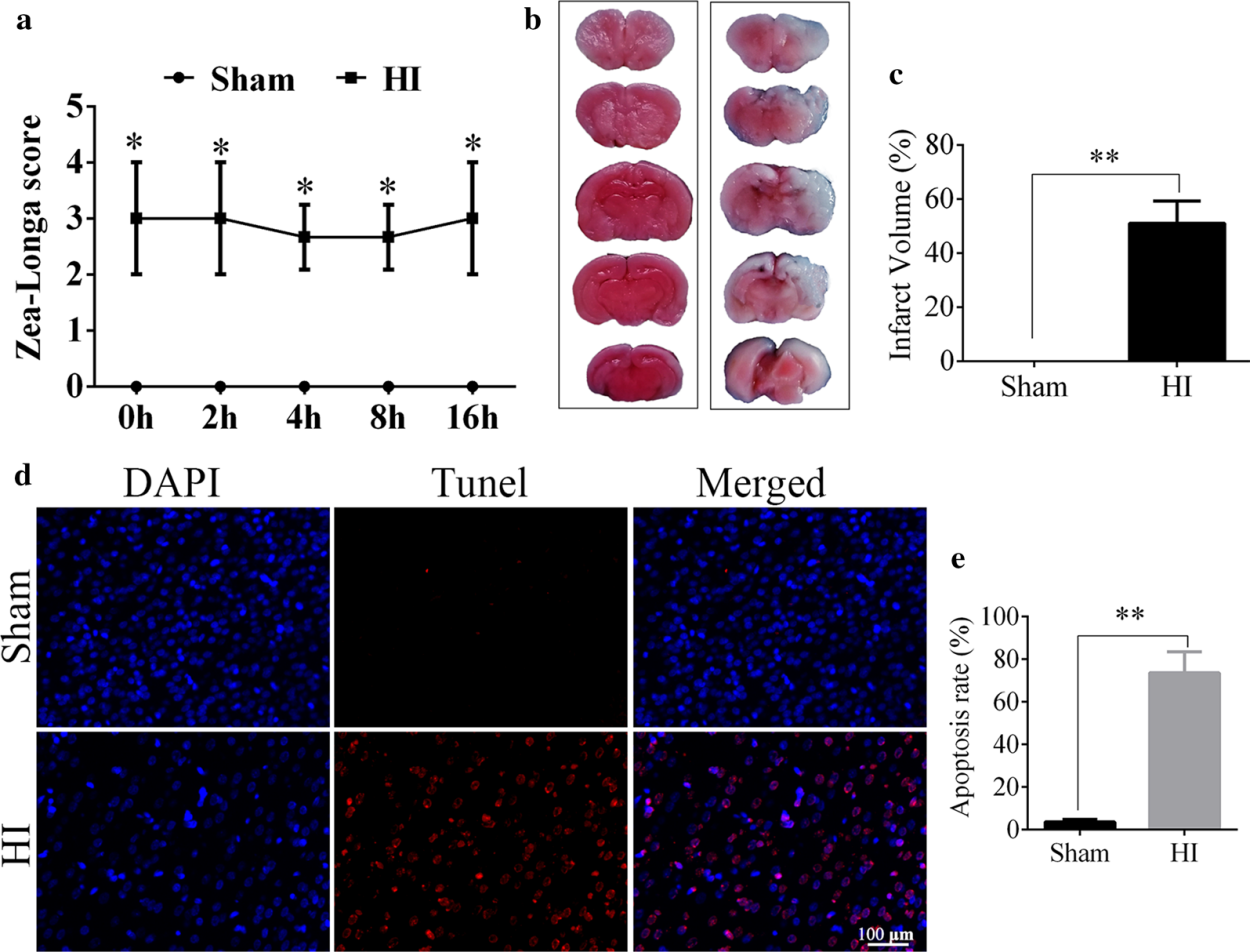
Successful establishing of HIE model in vivo. **a** The zea-longa scores in sham and HI groups. N = 20/group. **b** The comparison of TTC staining between sham and HI groups, and the white staining represented the ischemic area. N = 5/group. **c** The percentage of infarct area in sham and HI groups. **d** Tunel assay showed the apoptotic cells in right cortex. Red fluorescence represents the positive tunel staining, and blue is the nucleus staining with DAPI. N = 3/group. **e** Apoptosis rate in the right cortex, revealed by the tunel/DAPI. *HI* hypoxic-ischemic, *h* hour, *TTC* Triphenyte-trazoliumchloride, *tunel* terminal deoxynucleotidyl transferasedUtp nick end labeling staining. Data are presented as the mean ± SD. *p < 0.05, **p < 0.01. Scale bar = 100 μm

TTC staining and zea-longa scoring were used to evaluate the reliability of the HI model establishment in this study. The zea-longa test showed an obvious increase in the score of rats in the HI group compared with the sham group (HI vs. sham, *p* < 0.05, Fig. 1a). TTC staining showed that the infarcted brain tissue was white without detectable staining, while the unaffected brain tissue was stained red. Compared with the sham group, a quantitative analysis showed that the infarct volume in HI group was obviously higher than that in the sham group (HI vs. sham, *p* < 0.01, Fig. 1b, c), which further confirmed the HI model was established successfully. In addition, the TUNEL staining revealed that the apoptosis rate in the infraction region of the right cortex in the HI group was significantly increased in comparison with the sham group (HI vs. sham, *p* < 0.01, Fig. 1d, e).

### HI caused neuron loss

**Fig. 2:**
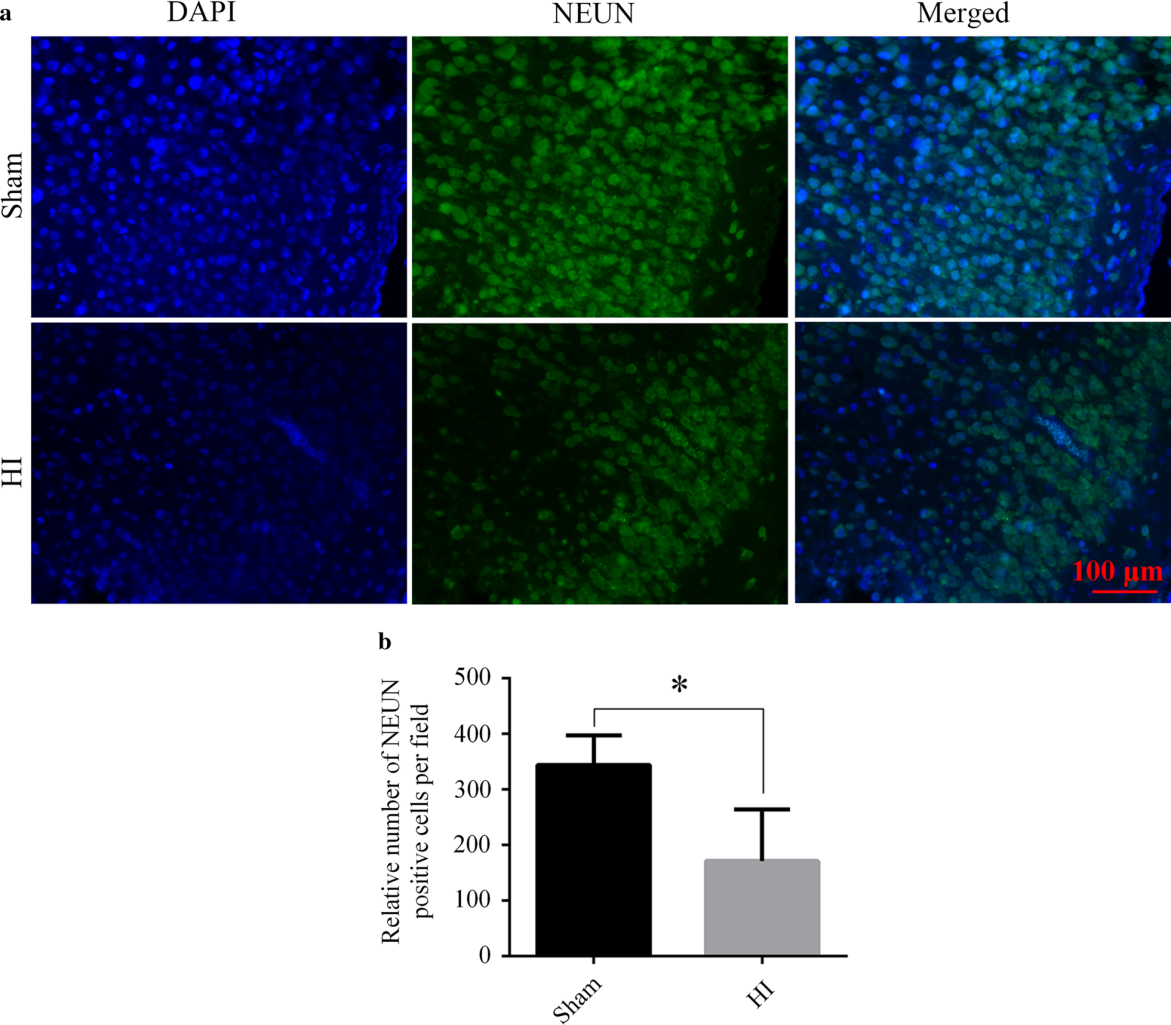
Evaluation of neuronal loss after HI. **a** Representative images of immunofluorescent staining of NeuN (a neuron marker) in the right cortex. Green staining represents the NeuN positive cells, and the nucleus is stained by blue. **b** Bar chart of relative number of NeuN positive cells per field. N = 3/group. HI: hypoxic-ischemic. Data are presented as the mean ± SD. *p < 0.05. Scale bar = 100 μm

Immunofluorescent staining showed that the nuclei of neurons were labeled with NeuN. The fluorescence intensity weakened and positive cells of NEUN specific for neurons decreased in the HI group, in comparison to sham group (HI vs. sham, *p* < 0.05, Fig. 2a, b).

### COX5A expression was decreased after HI injury in vivo and in vitro

**Fig. 3:**
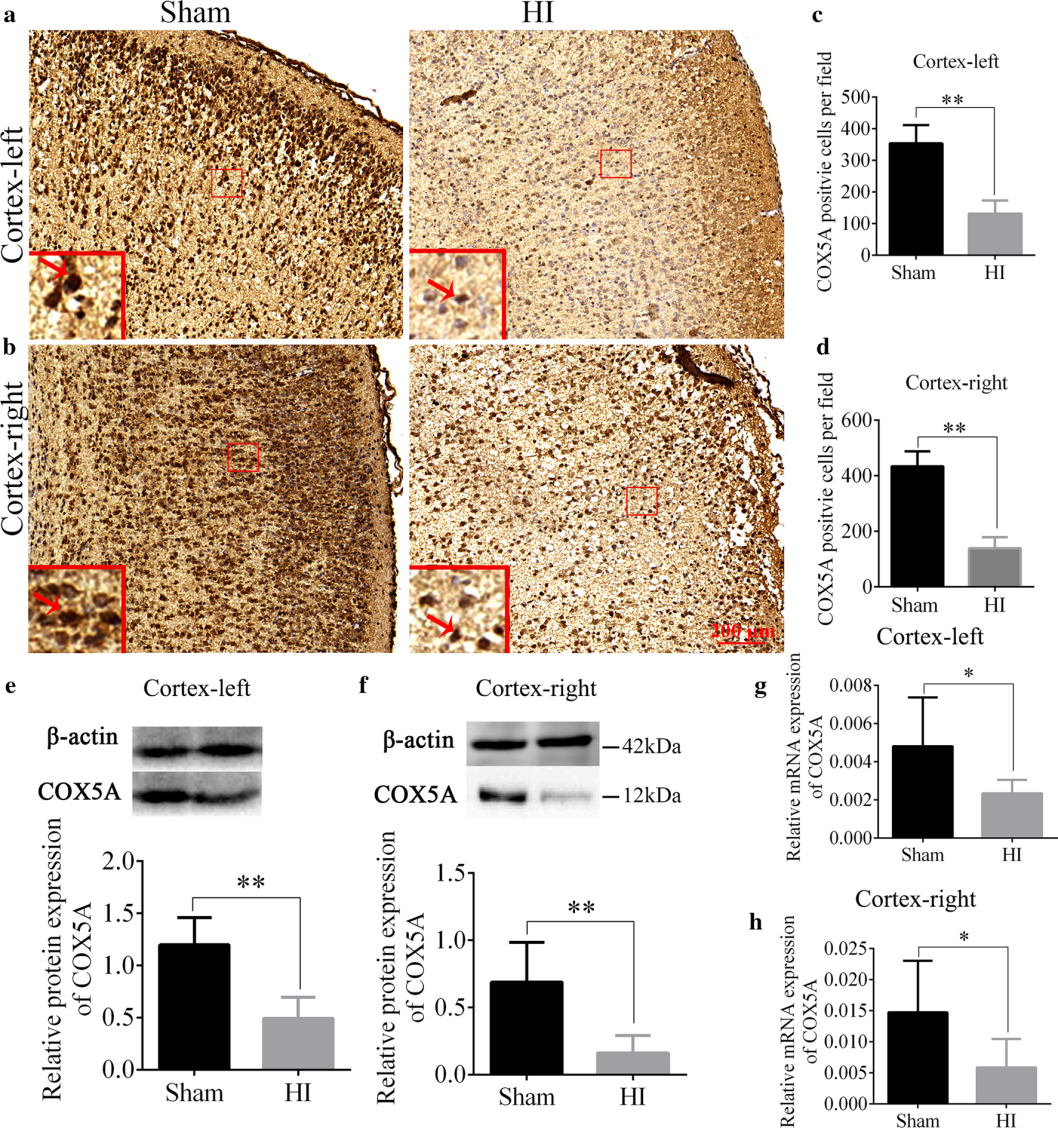
The expression of COX5A after HI. **a**, **b** Representative images of immunohistochemical staining of COX5A in left- and right-cortex, Red arrows represent the COX5A positive cells, which were stained by the dark brown colour. N = 3/group. **c**, **d** Bar charts of the COX5A positive cells in left- and right- cortex. N = 3/group. **e**, **f** WB results of the COX5A protein expression in left- and right-cortex. N = 6/group. **g**, **h** qRT-PCR results of the COX5A mRNA expression in left- and right-cortex. N = 6/group. *HI* hypoxic-ischemic, *COX5A* cytochrome c oxidase subunit 5a, *WB* western blotting, *qRT-PCR* quantitative real-time polymerase chain reaction. Data are presented as the mean ± SD. *p < 0.05, **p < 0.01. Scale bar = 200 μm

To detect the expression changes of COX5A in the cerebral cortex after HI, we performed immunohistochemistry, qRT-PCR and WB. The immunohistochemistry results showed that COX5A was localized in the nucleus of the cortex at 16 h (Fig. 3a, b). Compared with the sham group, the positive cells of COX5A were significantly reduced in the left and right cortex in the HI group (HI vs. sham, *p* < 0.01, Fig. 3c, d). COX5A protein and mRNA expression were significantly diminished in the left and right cortex as compared with the sham group revealed by WB (HI vs. sham, *p* < 0.01, Fig. 3e, f) and qRT-PCR detection (HI vs. sham, *p* < 0.05, Fig. 3g, h).

**Fig. 4:**
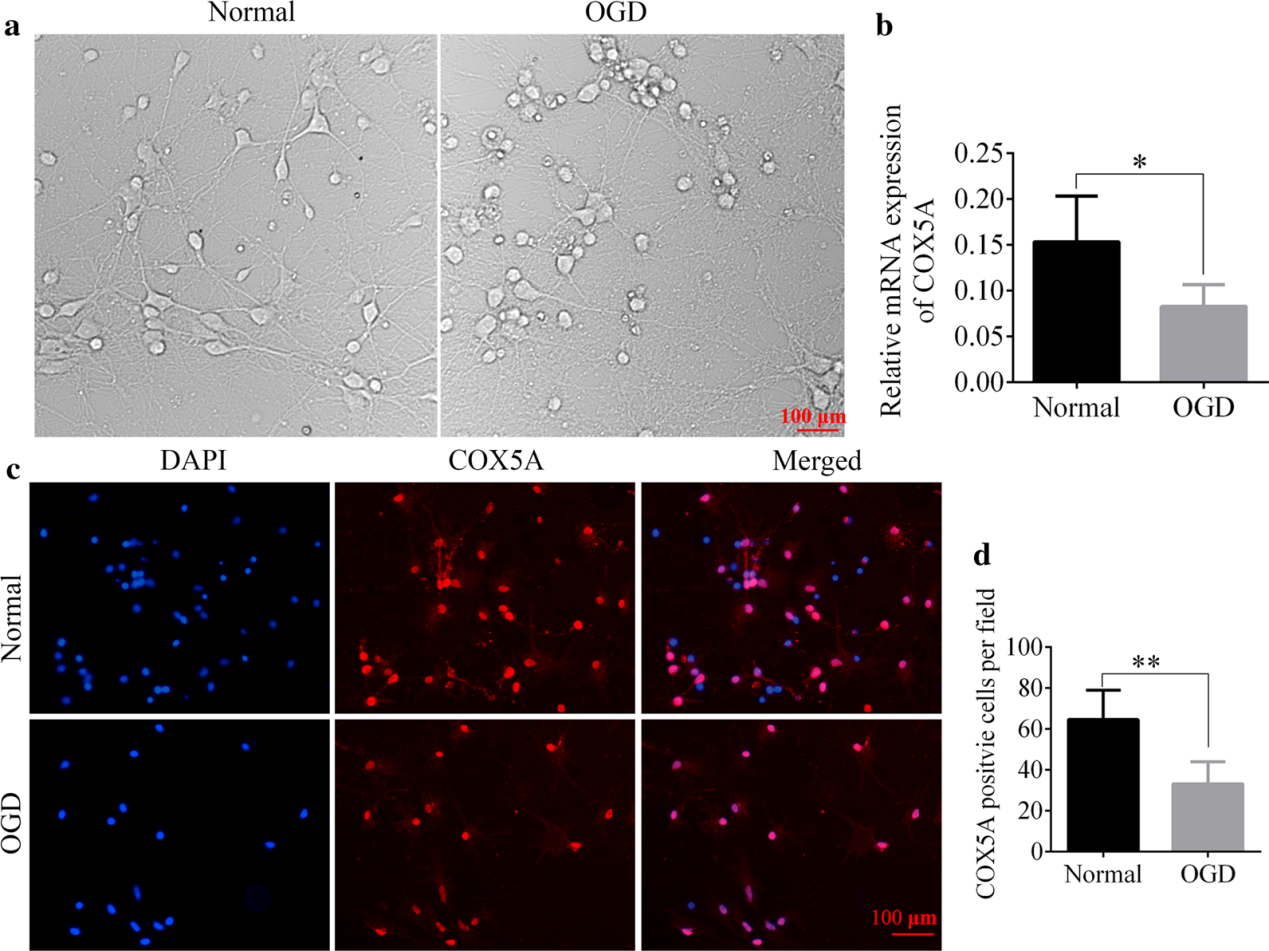
The expression of COX5A in OGD neurons. **a** The bright field of neurons in normal and OGD groups. Scale bar = 100 μm. **b** Quantitative analysis of relative mRNA expression of COX5A in neuron. N = 6/group. **c** Immunofluorescent staining of COX5A in normal and OGD groups. Red staining represents the COX5A positive cells, and the nucleus is stained by blue, n = 3/group. Scale bar = 200 μm. **d** The COX5A positive cells per field were calculated. N = 6/group. *OGD* oxygen–glucose–deprivation, *COX5A* cytochrome c oxidase subunit 5a, *qRT-PCR* quantitative real-time polymerase chain reaction. Data are presented as the mean ± SD. *p < 0.05, **p < 0.01

In the bright-filed images, we observed that compared with the normal group, differentiation and development of neurons was slower in the OGD group 90 min after OGD operation (Fig. 4a). In addition, COX5A mRNA expression was also decreased in the OGD cells (OGD vs. normal, *p* < 0.05, Fig. 4b). Moreover, immunofluorescent staining further revealed that the number of COX5A positive cells was decreased significantly in OGD group as compared with the Normal group (OGD vs. normal, *p* < 0.01, Fig. 4c, d).

### HSV-COX5A vector was successfully transducted in the primary neuronal cells

**Fig. 5:**
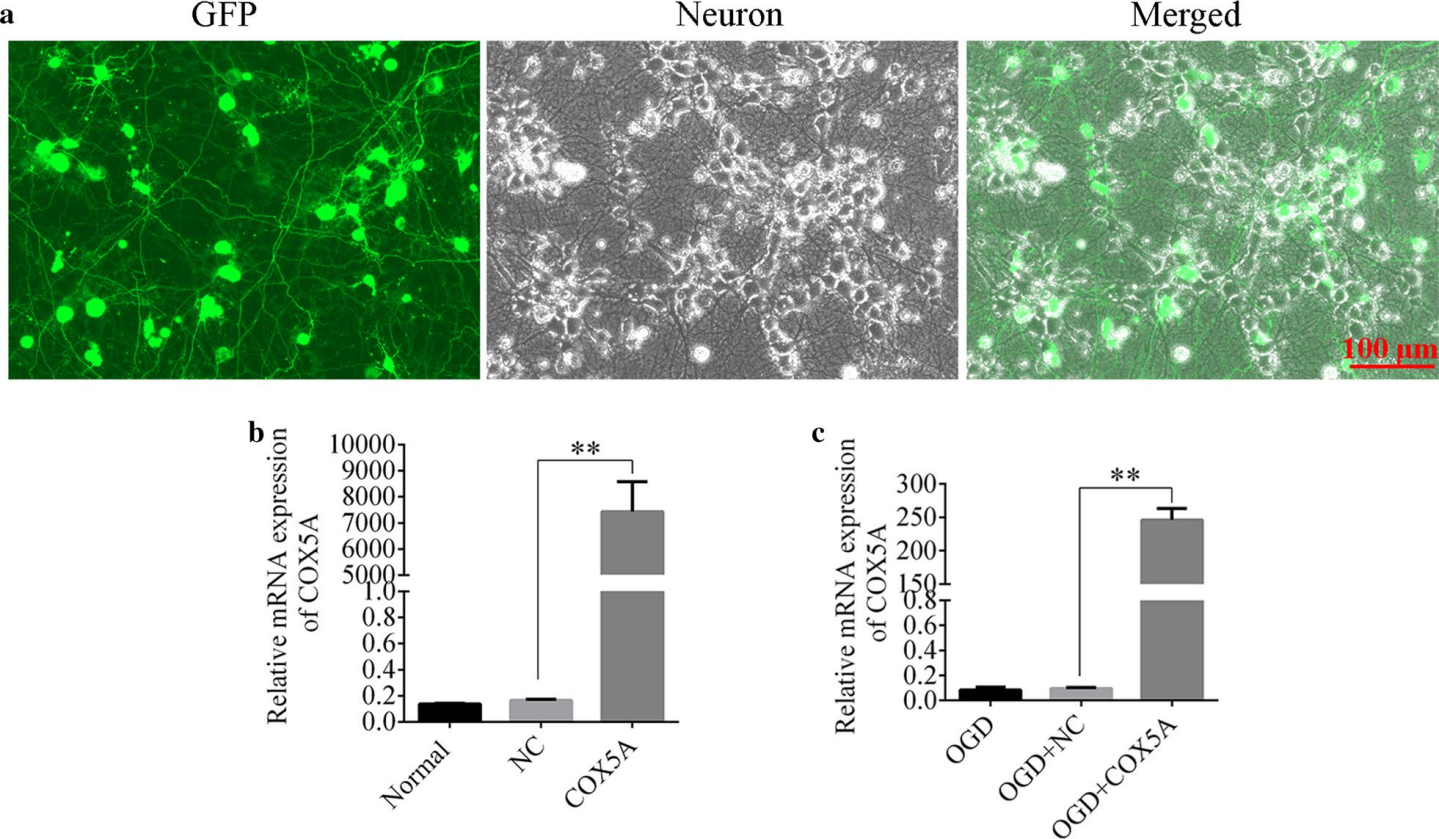
Transduction and verification of HSV contained COX5A over-expression. **a** The effective transduction of the HSV virus into neurons at MOI = 10. Green fluorecence corresponded to a green fluorescent protein which was a marker protein in HSV vector. Gray corresponded to neuron in the bright field with PH channel. N = 6/group. **b**, **c** Verification of COX5A expression using qRT-PCR in normal and OGD conditions, respectively. N = 6/group. *OGD* oxygen–glucose–deprivation, *NC* negative control, *GFP* green fluorescent protein, *COX5A-ORF* cytochrome c oxidase subunit 5a over-expression, Data are presented as the mean ± SD. **p < 0.01. Scale bar = 100 μm

The successful transduction of HSV-COX5A vector into the primary cortical neurons was demonstrated by the green fluorescence emitted by GFP in Fig. 5a, and the infection rate reached about 92%. What’s more, qRT-PCR showed that the mRNA expression of COX5A was significantly increased in the COX5A over-expression group in both normal and OGD conditions as compared with the NC (COX5A-ORF vs. NC, *p* < 0.01, Fig. 5b, c). It demonstrated that COX5A was successfully over-expressed in the cortical neurons.

### Over-expression of COX5A promoted the neuronal outgrowth and decreased the cell apoptosis

**Fig. 6:**
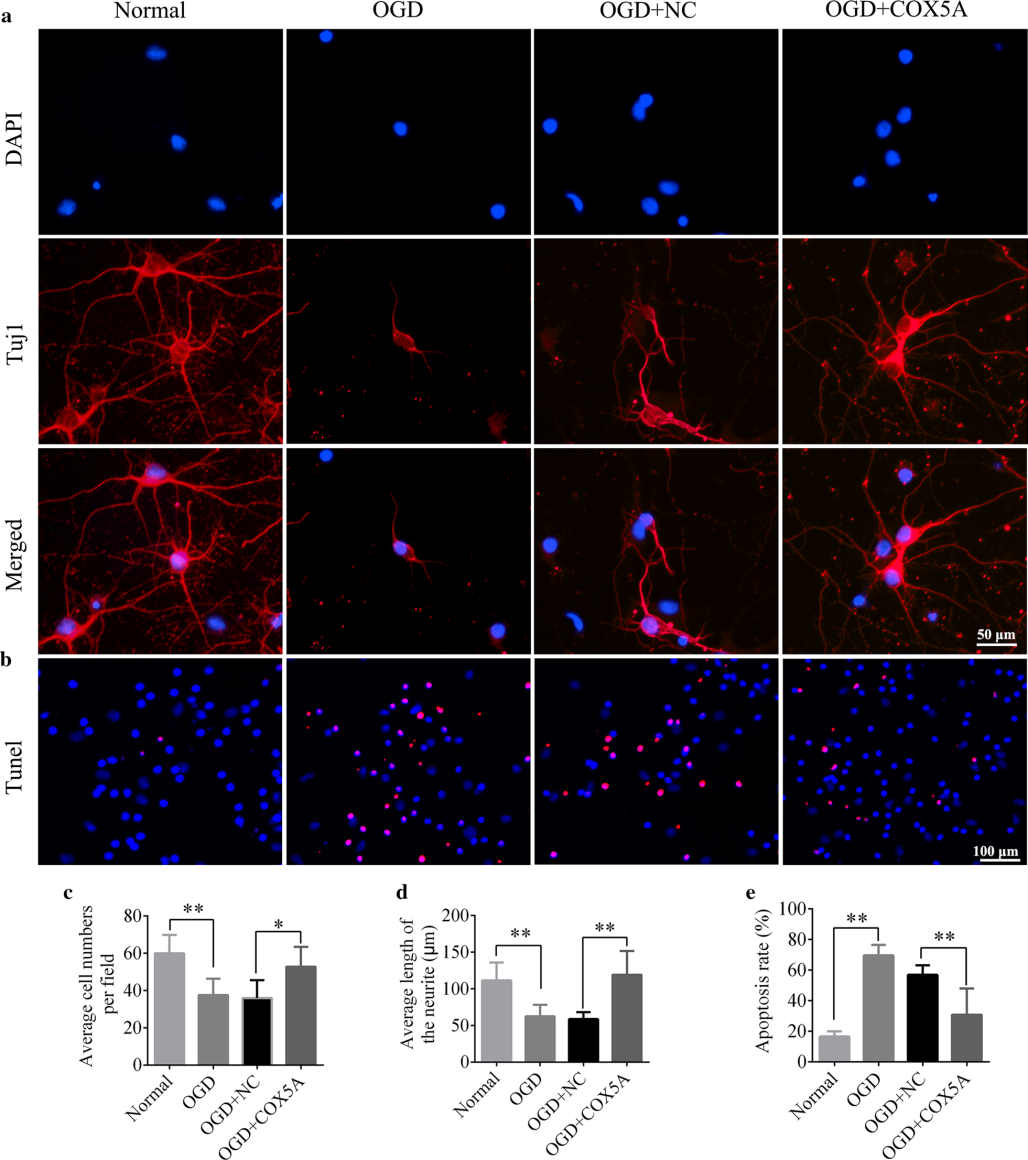
The role of COX5A over-expression on neurites outgrowth and cell apoptosis in vitro. **a** Representative images of Tuj1 immunofluorecent staining in each group, DAPI labeled nucleus (blue), Tuj1 labeled neurons (red). Scale bar = 50 μm. **b** TUNEL assay in neurons after COX5A over-expression under OGD condition. DAPI labeled nucleus (blue), red color represents positive apoptotic cells. Scale bar = 100 μm. **c** Quantitative histogram of average cell number per field. **d** Bar chart of average length of neuites. **e** The apoptosis rate of the neurons, which was indicated by tunel/DAPI. *OGD* oxygen–glucose–deprivation, *NC* negative control, *COX5A* cytochrome c oxidase subunit 5a over-expression, *tunel* terminal deoxynucleotidyltransferasedUtp nick end labeling. N = 6/group. Data are presented as the mean ± SD. *p < 0.05, **p < 0.001

To study the influence of COX5A overexpression on neurites outgrowth and neuronal apoptosis, immunofluorescent staining of Tuj1 and TUNEL assay were employed (Fig. 6a). Quantitative evaluation revealed that the OGD treated cells had overall shorter length of neurites and less cell number relative to normal group (OGD vs. normal, *p* < 0.01, Fig. 6a, d, e). In addition, OGD also led to increased cell apoptosis indicated by the elevated apoptosis rate (OGD vs. normal, *p* < 0.01, Fig. 6b, f). However, COX5A over-expression in OGD neurons showed a significant elevation in the cell number, and increase in the neurite lengths as well as decrease in apoptosis rate in comparison with the OGD + NC group (OGD + COX5A vs. OGD + NC, *p* < 0.05, *p* < 0.01, Fig. 6d–f). These revealed COX5A over-expression could maintain the neurites and inhibit neuronal apoptosis so as to improve neurological function.

### COX5A over-expression markedly up-regulated TPI expression

**Fig. 7:**
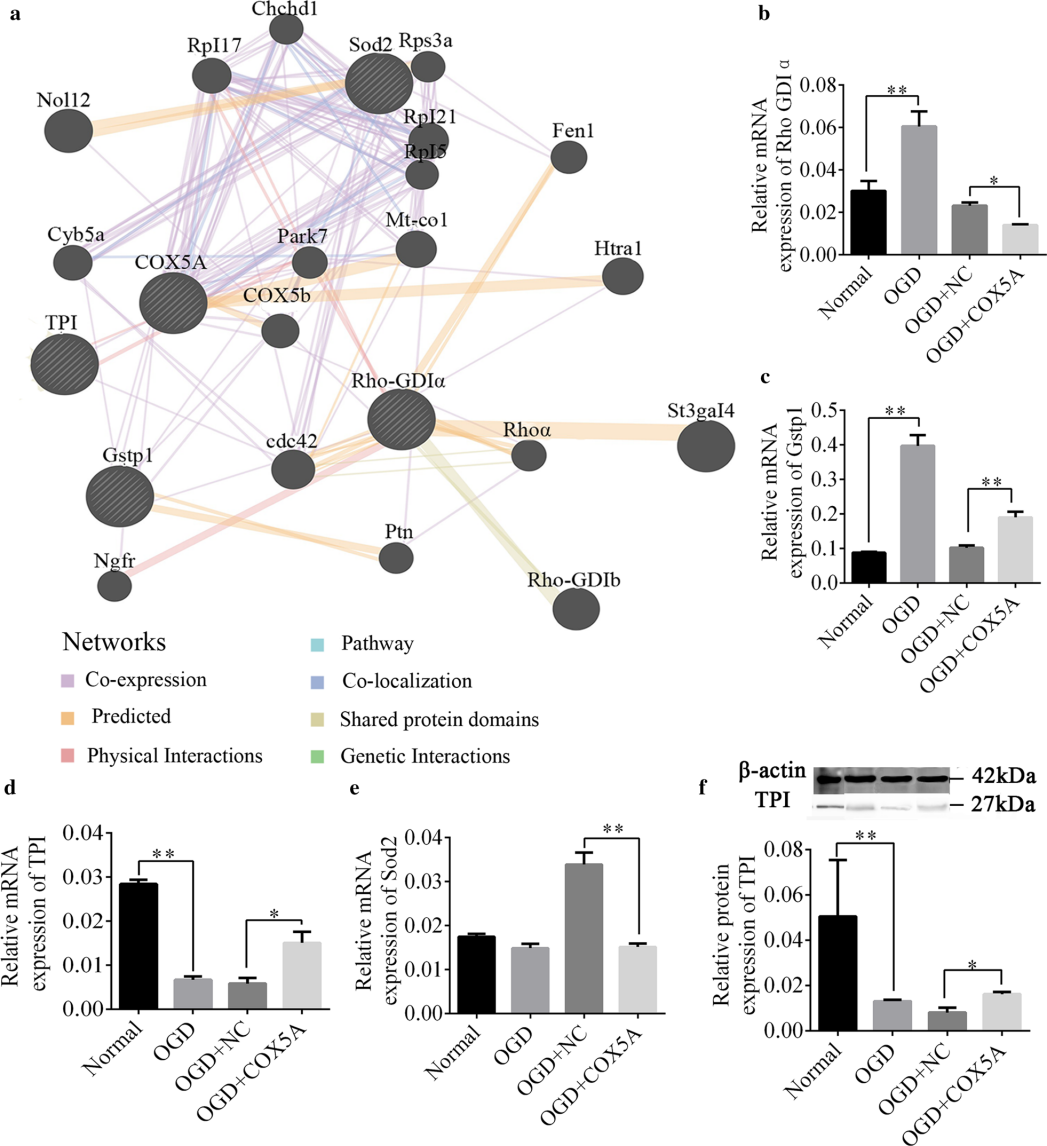
The molecular mechanism of COX5A over-expression on neurite outgrowth after OGD injury. **a** The relationship between Gstp1, Sod2, Rho-GDIa, TPI and COX5A predicted by GeneMANIA. **b** The mRNA expression of Rho GDP-dissociation inhibitor1. **c** The mRNA expression of Gstp1. **d** The mRNA expression of TPI. **e** The mRNA expression of Sod2. **f** The protein expression of TPI in each group. *OGD* oxygen glucose deprivation, *NC* negative control, *COX5A* cytochrome c oxidase subunit 5a over-expression, *Gstp1* glutathione *S*-transferasp1, *Sod2* Superoxide dismutase 2, *Rho-GDIα* guanine dissociation inhibitor α, *TPI* triosephosephate isomerase. N = 6/group. Data are presented as the mean ± SD. *p < 0.05, **p < 0.001

GeneMANIA was used to explore the underlying molecular mechanism of COX5A over-expression in regulating neurites outgrowth and cell apoptosis in the cortical neurons. Interestingly, four genes (Gstp1, TPI, Rho GDIa and Sod2) were screened out. What’s more, it was found that Gstp1 and TPI are co-localized with COX5A, and the TPI was physically interacted with COX5A (Fig. 7a). Moreover, the qRT-PCR verification showed that as compared to the normal group, the mRNA levels of Rho-GDIa and Gstp1 were markedly increased while the expression of TPI was reduced in OGD group, and Sod2 levels didn’t respond to the OGD (OGD vs. normal, *p* < 0.01, Fig. 7b–e). Conversely, in the OGD cells over-expressing COX5A, the mRNA expression of Rho-GDIa and Sod2 was significantly reduced (OGD + COX5A vs. OGD + NC, *p* < 0.05, Fig. 7b, e), while the Gstp1 and TPI expression was significantly increased (OGD + COX5A vs. OGD + NC, *p* < 0.01, Fig. 7c, d). Altogether, the findings revealed that only TPI expression was positively correlated with COX5A. WB analysis further confirmed the protein expression of TPI was increased after COX5A overexpression in OGD neurons (OGD + COX5A vs. OGD + NC, *p* < 0.05, Fig. 7f).

## Discussion

In this study, employing a rat model with neonatal hypoxic-ischemic in vivo, and OGD neuronal cell injury model in vitro, we found that the expression of COX5A was significantly decreased after HI with more neuronal damages and apoptosis in the right cortical injury area. In addition, over-expression of COX5A effectively promoted the outgrowth of neuronal neurite and reduced apoptosis in neurons subjected to OGD, and the potential molecular mechanisms are closely related to the up-regulation of TPI expression. This may provide a new idea for future clinical treatment with HI injury.

### The HI model was successfully established in P7 rats

In this study, the neonatal HI model was successfully established based on the classic Rice-Vannucci method of neonatal HI [23, 24]. Previously, an MRI study compared the HI model by Rice-Vannucci and the neonatal stroke filament occlusion, which revealed that the neonatal stroke injury is restricted in the middle cerebral artery, while it spreads collaterally in the Rice-Vannucci HI model [29] Therefore, the Rice-Vannucci model of neonatal HI has been used the most in the basic study. The zea-longa scores were used to evaluate the neurological function in ischemic model, and also applied to verify the hypoxic-ischemic model establishment in neonatal rats [30, 31], Moreover, literature proved that the brain injury of P7 rats equals that of full-term or near-term human fetuses [32]. Additionally, P7 rats represent the peak brain growth, which occurs at term humans and is equivalent to 34 weeks’ gestation [33]. Therefore, HI model in the present study was established in P7 neonatal rats by the right common carotid artery ligation and subsequent hypoxia for 2 h. As a result, the cerebral injuries were mainly concentrated in the right side of the brain [24], thus, we focused on the right cerebral hemisphere in the later observation of brain damage.

### Decreased expression of COX5A induced the neuronal injury

In the present study, the expression of COX5A was decreased after HI injury. Multiple studies [34–37] reported that the expression of COX5A decreased in a variety of central nervous system diseases, which in turn caused an imbalance in neuronal energy regulation. Moreover, Wei HL reported that down-regulation of COX5A seriously impaired the sensory function in a neuroplastic model of SD rat after dorsal root ganglion resection [38]. What’s more, the down-regulation of COX5A led to mitochondrial damage and dysfunction, further accelerated disease progression in the course of HIE disease [39, 40]. COX5A reduction has also been demonstrated close correlation with acute myocardial infarction [41] and diabetes [42]. However, little information has been reported about the role of COX5A in neuronal growth after SCI injury. Exactly in this study, we demonstrated the cell number reached about 60 per field and the each neurite length exceeded 100 μm under normal condition, which were conversely reduced a lot in OGD neurons. Nevertheless, the decreased trend was significantly reversed in OGD neurons when overexpressing COX5A. These indicated the decrease of COX5A may induce the neuronal damage and cell apoptosis, while overexpression of COX5A could promote the neurites length and depressed neuronal apoptosis, strengthening the crucial role of COX5A in the process of HIE.

### Over-expressing COX5A for neuronal improvement is associated with TPI up-regulation

Our study found that the over-expression of COX5A promoted the outgrowth of neurites and reduced apoptosis in neurons subjected to OGD, therefore uncovering its crucial role in the recovery of HIE. As for the role of COX5A, a previous study found that the COX5A expression was up-regulated in the injured area after acupuncture treatment with rats after spinal cord injury. Besides, XiYang YB constructed COX5A over-expression transgenic mice and found that hippocampal neurons have enlarged cell bodies and increased dendritic complexity, and hippocampal-dependent spatial learning and memory ability got improved [43]. These indicated that increasing COX5A expression may exert neuroprotective effect on the central nervous system diseases. Similarly, our study found that the expression of COX5A was reduced in HIE rats and OGD neurons, and transduction of HSV-COX5A over-expressing virus into cortical neurons in vitro reduced apoptosis and increased neuronal neurite lengths to alleviate the OGD injury, therefore supporting the positive role of COX5A in neural repair after HI. For the mechanism of COX5A, it was well-established that COX5A over-expression could increase CGRP densities in lamina of the spinal cord [44] to further promote wound healing. Whereas, the present study concealed that the over-expression of COX5A could markedly up-regulate the TPI level in OGD neurons, which suggests TPI may be the downstream targets of COX5A in the HI process. As a glycolytic enzyme [45], mutations of TPI in coding gene revealed its association with neurological phenotypes [46]. Also, TPI deficiency is associated with progressive neurological dysfunction and commonly leads to the death of the carrier in early years of childhood [47]. Recently, through development of genetically engineered variants of TPI, it was hypothesized that this enzyme has non-catalytic function that is important for the survival of neurons, which explains the neurological complications associated with TPI deficiency [48]. Nevertheless, TPI is identified as a target for arginine methyltransferase 5 and cyclin dependent kinase 2 [49, 50]. These findings demonstrate the strict regulation of TPI by cell cycle pathways, and explain their neurological importance. In fact, new evidence showed the firm relationship between cell cycle imbalance and neurological disorder [51], in which, Alzheimer’s disease is considered one example where failure in cell balancing results in massive neurodegeneration [52]. Together, TPI up-regulation in this study could be useful to improve neurological function in the HIE insults, and its role is positively correlated with COX5A.

## Conclusion

Collectively, we demonstrated the decrease of COX5A in the cortex after HI and OGD injury, whereas, over-expressing COX5A in OGD condition could largely improve cell function. The possible mechanism is associated with TPI up-regulation. These findings may contribute to the mechanism explanation of HIE and provide new idea for the treatment of HIE in future clinic trial.

## Data Availability

The datasets analyzed during the current study available from the corresponding author on reasonable request.

## Abbreviations

OGD: Oxygen–glucose–deprivation
NC: Negative control
COX5A: Cytochrome c oxidase subunit 5a over-expression
Gstp1: Glutathione *S*-transferasp1
Sod2: Superoxide dismutase 2, Rho-GDIα: Guanine dissociation inhibitor α
TPI: Triosephosephate isomerase
TUNEL: Terminal Deoxynucleotidyl TransferasedUtp Nick End Labeling
TTC: Triphenyte-trazoliumchloride
HI: Hypoxic-ischemic
WB: Western blotting
qRT-PCR: Quantitative real-time polymerase chain reaction
GFP: Green Fluorescent Protein
